# Extensive Sequestration Chronic Maxillary Osteomyelitis in an Uncontrolled Diabetic Patient: Comprehensive Case Management of a Rare Entity

**DOI:** 10.1055/s-0043-1771536

**Published:** 2023-10-17

**Authors:** Prasiddha Mahardhika El Fadhlallah, Andreas Pratama Nugraha, Okky Prasetio, Indra Mulyawan

**Affiliations:** 1 Oral and Maxillofacial Surgery Specialist Program, Faculty of Dental Medicine, Airlangga University, Surabaya, Indonesia; 2 Department of Oral and Maxillofacial Surgery, Dr Mohamad Soewandhie General Hospital, Surabaya, Indonesia; 3 Department of Oral and Maxillofacial Surgery, Faculty of Dental Medicine Universitas Airlangga, Surabaya, Indonesia

**Keywords:** sequestration, osteomyelitis, maxillary, comprehensive, management, diabetes mellitus

## Abstract

The hallmark of osteomyelitis was progressive bone destruction and sequestrum formation. In the underlying disease, like diabetes mellitus, osteomyelitis becomes severe and exacerbates the condition. It was essential for the oral and maxillofacial surgeon to comprehend its complex medical and surgical management to achieve complete disease eradication. The aim of this article was to report a rare case and comprehensive management of extensive sequestrating maxillary osteomyelitis with uncontrolled diabetes mellitus patients. A 58-year-old male patient with pain and swelling accompanied by discharge of pus in the sinistra maxilla region. The systemic disease was identified as uncontrolled diabetes mellitus, and had a history of unhealing wounds 1 year ago after upper left molar extraction. Computed tomography scan result showed extensive sequester formation and bony destruction in the right extending to the left maxilla. Microbial culture results were *Klebsiella pneumoniae* and *Morganella morganii.* Subsequently, extensive sequestrectomy and multiple extractions of the involved jaw and teeth were performed after diabetes mellitus was regulated. A suspension suture against oral and nasal mucosa was performed to avoid dead space formation. Comprehensive perioperative management in maxillary osteomyelitis in uncontrolled diabetes mellitus includes sequestrectomy, definitive antibiotic therapy based on culture results, and diabetes regulation to improve the successful management of this case.

## Introduction

Osteomyelitis comes from the ancient Greek words *osteon* (bone) and *muelinos* (marrow) and describes a bone tissue infection that includes the marrow or bone cortex caused by polymicrobial infection. 1 Osteomyelitis also results in inflammatory conditions of the bones involving the Haversian canals and widespread into the periosteum. 2 Most cases of osteomyelitis occur in the fifth and sixth decade of age. In general, patients come already in an advanced stage and are accompanied by uncontrolled comorbidities, 3 like diabetes mellitus. Diabetes mellitus probably cause recurrent osteomyelitis. 4

Clinically, the osteomyelitis resemblance to bisphosphonate-related osteonecrosis of the jaw. But this condition, it can be distinguished from anamnesis in patients with no history of bisphosphonate consumption and no history of radiation therapy in the jaw area. 5 6

The case of maxillary osteomyelitis was rare, 3 7 8 9 and some of the reports were accompanied by actinomycosis, 10 11 12 and mucormycosis, 13 14 15 16 17 but one study reported a higher prevalence than mandibular. 8 The incident rate in mandibular is around 66.7%, 18 but the maxilla is only 33.3%. 8 The maxilla is a jaw bone with good vascularity and thin cortical bones and makes not susceptible to infection. 19 20 For this reason, the prevalence of maxillary osteomyelitis is low. Many cases of osteomyelitis were obtained in the mandibles mainly due to odontogenic infections exacerbated by immunocompromise and uncontrolled metabolic diseases such as diabetes mellitus, acquired immunodeficiency syndrome, and malnutrition. 8 21 The maxillary osteomyelitis in diabetes mellitus patients also rarely reported. 4 22 23 Diabetes mellitus become a risk factor for the severity of osteomyelitis, 24 and secondary infection. 25 In India, the prevalence rate of maxillary osteomyelitis with diabetes mellitus is 45.1%. 26 This case report aims to present maxillary osteomyelitis with diabetes mellitus and discuss aspects of diagnosis enforcement and comprehensive management.

## Case Presentation

### Chief Complaint

A 58-year-old male patient came to Oral Surgery Department, at Mohammad Soewandhie Hospital with complaints of pain in the upper left cheeks and gums after tooth extraction of the upper jaw in January 2021. After extraction, he complained of pain and swelling accompanied by pus discharge in the extraction area. The patient still feels pain and pus often comes out from the exposed bone. The patient had medical history of uncontrolled diabetes mellitus since 2010 and got treated with glimepiride, metformin, and acarbose drugs. There was no history of chemotherapy or radiotherapy treatment and no consumption of bisphosphonate drugs.

### Extraoral and Intraoral Examination

**Fig. 1:**
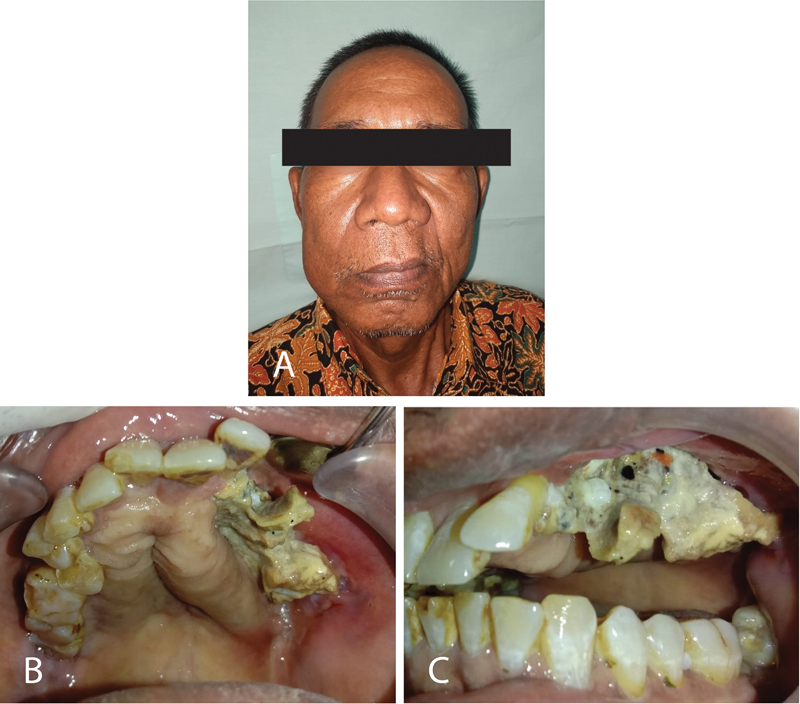
The extraoral examination revealed an asymmetrical area of the sinistra of the maxilla ( **A** ). The palate durum was pressed toward medial ( **B** ). The bone exposed in the buccal palate on regions 23 to 28 and 14 to 15 ( **C** ).

Extraoral examination in the maxillofacial region showed facial asymmetry and the swelling on maxillary sinistra. On palpation, there was palpable asymmetry, concavity was felt on the left maxilla, and there was no maxillary bone and paresthesia in the infraorbital area ( Fig. 1A ).

Intraoral examination revealed bone exposed buccal-palate region of 23 to 28 and 14 to 15. Palatum durum on the sinistra was pressed toward the medial and soft consistency. The region of 27 was observed as gangrene radixes and hyperemic ( Fig. 1B ).

On palpation in the region 23 to 28, the area was rough and sharp surface, hard consistency, and moveable, indicating a bone segment ( Fig. 1C ).

### Radiograph Examination

**Fig. 2:**
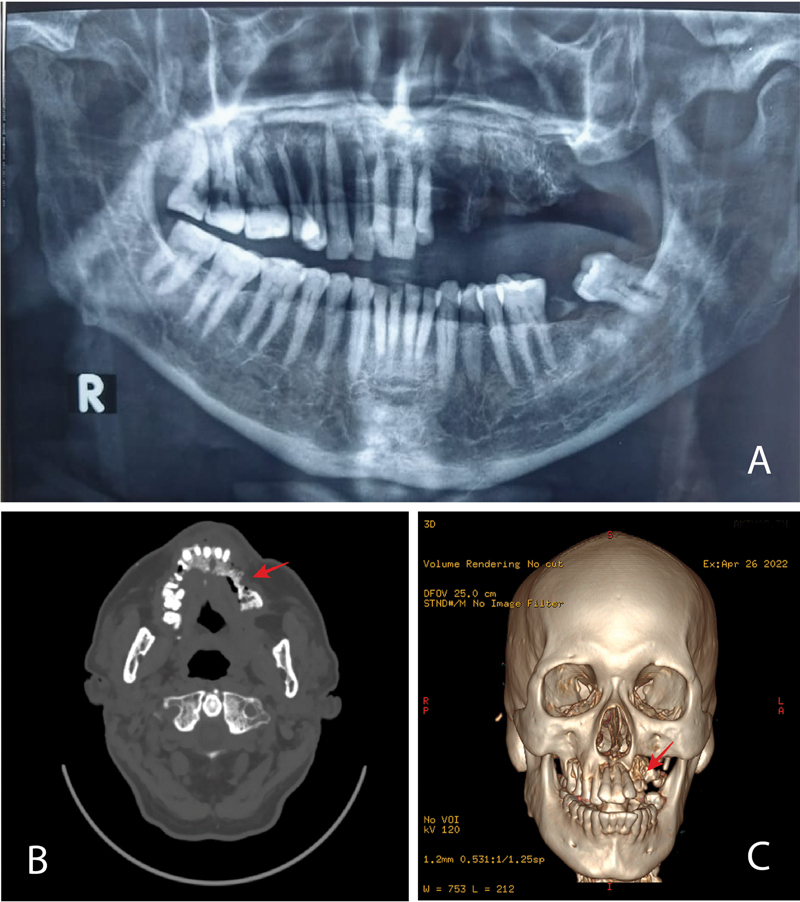
Panoramic radiography ( **A** ). Computed tomography (CT) scan examination (bone window) axial view ( **B** ); The three-dimensional CT scan looks frontal and axial ( **C** ). The red arrow shows a picture of bone destruction.

The panoramic radiography and computed tomography (CT) scan were carried out. On the panoramic radiography, there were multiple radiopaque indicated as *moth-eaten appearance* in the sinistra maxilla region 21 to 28 ( Fig. 2A ). The CT scan examination showed a radiopaque mass in the sinistra maxilla region 21 to 28 and extending to the dextra maxilla ( Fig. 2B ), possibly also hitting the left temporal base and the lower wall of the left maxillary sinus ( Fig. 2C ).

### Complete Blood Count

A blood examination was also carried out. The value of hemoglobin A1c (HbA1c) (12.9%) indicated uncontrolled diabetes. Other parameters, like hemoglobin (10%) and lower mean corpuscular volume and mean corpuscular hemoglobin concentration, indicated microcytic hypochromic anemia with suspicion of nutritional and iron deficiency. Of this finding, the patient was also referred to internal medicine to optimize the condition.

### Microbial Test

A puss was collected for the sinistra maxilla, and a microbial culture was performed. The result showed a dominance of *Klebsiella* *pneumoniae* and *Morganella morganii* .

## Case Management

Chronic suppurative osteomyelitis maxilla is a preoperative diagnosis based on anamnesis, clinical examination, and patient support examination results. The patient was previously referred for consultation and blood glucose level regulation and given the drugs glimepiride, metformin, acarbose, and folic acid by an internal medicine specialist to a random blood sugar below a value of 200 mg/dL. The patient was found to have a cardiac risk index—class I and was deemed eligible for oral surgery after being assessed by a cardiology specialist.

**Fig. 3:**
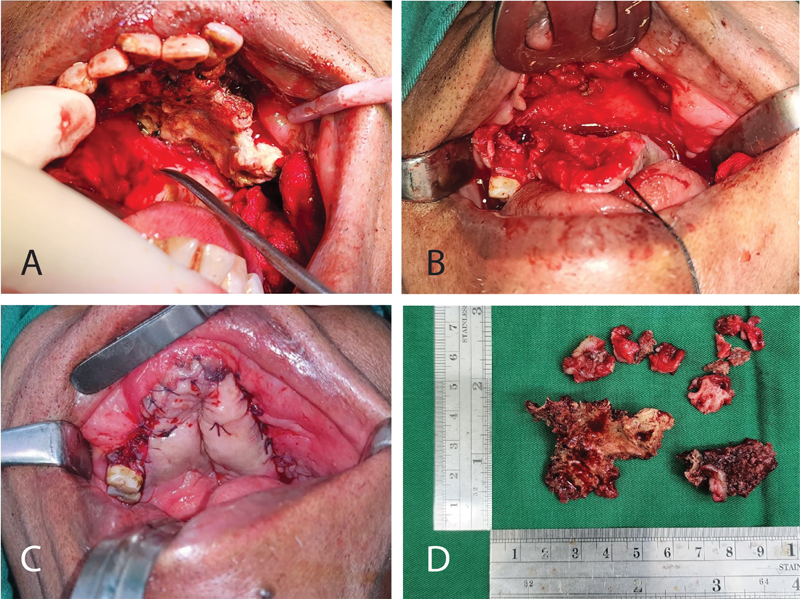
A mucoperiosteal flap of the maxillary region showed a necrotic bone ( **A** ). Postsequestrectomy defects appear that nasal mucosa is still indented ( **B** ). Postoperative intraoral mucosal suturing ( **C** ) and sequestrum specimens ( **D** ).

The surgery was carried out; in this case, an extensive sequestrectomy was performed. A mucoperiosteal flap incision was performed on the buccal and palatal regions 28 to 16 ( Fig. 3A and B ). Necrotic bone appeared in the mobile maxillary segment. The buccal section, bone resorption, and third-degree tooth shake were obtained, and then 22 to 16 tooth extraction, squashing, and smoothing of the bone surface were carried out ( Fig. 3D ). Postsequestrectomy defects of the nasal mucosa appeared to be still indented. The obturator could be installed in this case because there were no bones and teeth left, and it was adequate for retention; so suturing was carried out from the oral mucosa to the nasal mucosa to avoid the occurrence of dead space ( Fig. 3C ).

Bleeding during surgery was anticipated by preparing one bag of whole blood transfusion. Before the surgery, 1.5 g cefuroxime was administered to the patient. Following the surgery, a 1 g ceftriaxone injection was given based on the results of antibiotic sensitivity obtained from a 5-day torture of pus culture. The patient was then instructed to take cefixime 200 mg orally for 5 days as continued antibiotic therapy. Furthermore, an anti-inflammatory treatment of 30 mg ketorolac injection was given.

### Histopathology Diagnosis

**Fig. 4:**
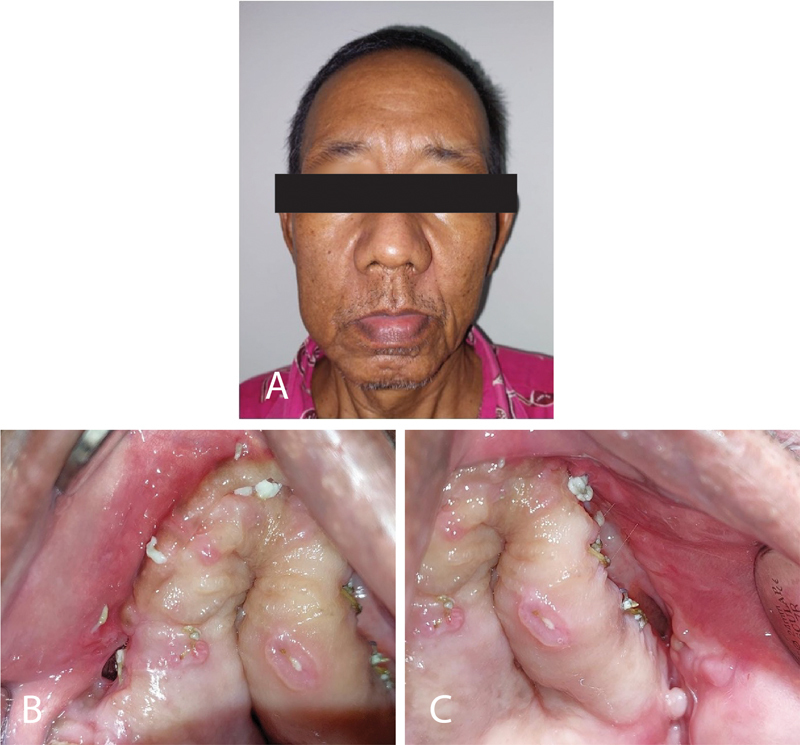
The result of surgery after a 1-month follow-up. The wound is healed and closed, and no bone is exposed.

The sequester was found to be positive for osteomyelitis based on the histopathological examination results. The tissue is partially coated by squamous epithelium, a solid density of inflammatory lymphocyte cells, plasma cells, neutrophils, and proliferation ( Fig. 4A ). Blood vessels appeared between the stroma (yellow arrow) of fibrous tissue and the fat matrix, and no sign of malignancy was obtained ( Fig. 4B ).

### Postsurgery Follow-Up

**Fig. 5:**
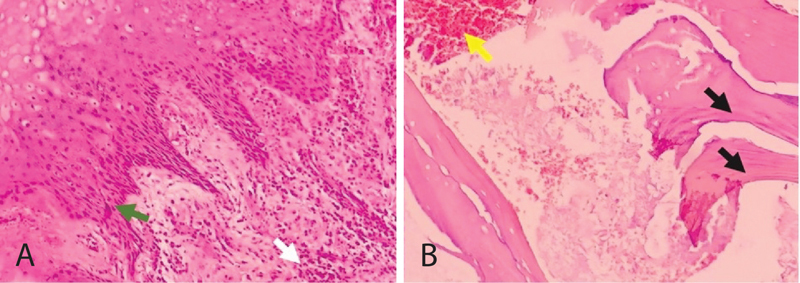
Microscopic picture examination of anatomical pathology (magnification 200x). The tissue is partially coated by squamous epithelium (green arrow) and a solid density of inflammatory cells (white arrow) ( **A** ). The necrotic part of the bone is characterized by the absence of living osteocytes and contains mostly mononuclear lymphocyte cells. A blood vessel (yellow arrow) appeared between the stroma and bone matrix (black arrow) ( **B** ).

Patients are instructed to a high-calorie, high-protein liquid diet for 6 days postsurgery. The patient was instructed to follow up, 1 month after discharge from the hospital for postoperative deflection area inspection. There was no abnormality observed both extra and intraoral ( Fig. 5 ).

## Discussion

Maxillary osteomyelitis is rare due to the extensive vascular supply; so, it is not easy to experience ischemia and necrosis in the maxillary region. 3 Typical clinical findings include pain, swelling, and odorous secretions. Microbiological etiology is by gram-positive and gram-negative microorganisms, including *Staphylococcus aureus, Staphylococcus epidermidis, Pepto streptococcus, Pneumococcus, Hemolytic streptococci, Escherichia coli* , and *Bacteroides* . 27 Osteomyelitis is classified into suppurative and nonsuppurative. Suppurative osteomyelitis is primarily caused by odontogenic infections characterized by fistulas and sequestration. 28

The case of maxillary osteomyelitis is rarely reported. The osteomyelitis is also found with the coinfection with *Mucorales* and *Aspergillus* species. 29 The diabetes status becomes a severity factor for osteomyelitis, especially in the maxilla. 4 Commonly seen in immunocompromised patients, diabetes mellitus is an important associated comorbidity in the pathophysiologic development of this disease. 30 Noncognizance of diabetes mellitus can be devastating for the maxillofacial region and may be fatal for the patient. 22 In another case, diabetes mellitus is able to trigger the exacerbation of osteomyelitis. 31

The incidence ratio of men to women in osteomyelitis can vary up to 5.2:1. In previous studies, the ratio of men to women was 1.1:1. The age of patients ranges from 40 to 70 years, with an average age of 51.6 years. The predilection ratio of osteomyelitis in the mandible and maxilla is 2:1. 23 32 It aligns with the patients involved, a 58-year-old male. The radiographic examination of chronic osteomyelitis is mainly in the form of *a* “moth-eaten appearance,” which occurs due to the expansion of the medullary space of the canal and the dilation of the Volkmann canal caused by intramedullar inflammation that continues into the bone ischemia of the lysis bone and granulation tissue surrounds a necrotic bone referred to as a sequester. 3 In this case, the sequester was also observed and led to chronic conditions. Through a CT scan, it confirmed that the sequesters extended to the maxillary region. Osteomyelitis is characterized by necrotic bones with irregular trabeculae bone formations that appear empty due to the absence of osteocytes, an osteoblastic layer, and inflammatory cells such as lymphocytes. The morphological picture of chronic osteomyelitis is the presence of a part of the bone that the absence of living osteocytes characterizes necrosis. Most contain mononuclear lymphocyte cells, granules, and fibrous tissue replacing bones absorbed by osteoclasts. 33

The main predisposing factors are impaired immunity disorders such as immunodeficiency syndrome, diabetes mellitus, autoimmune conditions, malignancies, and malnutrition. 12 According to Peravali et al, 68% of upper jaw osteomyelitis cases are associated with diabetes mellitus because in hyperglycemic conditions, the immune system decreases and results from impaired distribution of vascularization to the maxilla. 23 Previous study stated that the percentage of osteomyelitis in the maxilla was higher with uncontrolled diabetes (33.3%) compared to controlled diabetes (9.5%). 8 Diabetes mellitus is known as an immune response suppressor, 27 and has a strong correlation with osteomyelitis. 32 This correlation was proven in previous studies where diabetes was found to be one of the main factors for the occurrence of osteomyelitis (47.6%). The increased blood glucose levels weaken and damage the walls of capillary blood vessels that supply nerve nutrients. On the other hand, diabetic patients experience decreased chemotaxis function in leukocytes, phagocytosis, and leukocyte life span, causing tissue inflammatory response decreases. The reduction in the immune response is caused by impaired glucose metabolism and causes a long-wound healing process. 26 In this case, the patient has had a history of diabetes since 2010, and the HbA1c test indicates that the diabetes is uncontrolled. The patient also underwent an extraction of the upper jaw teeth in January 2021 and experienced persistent pain and healing wounds, which may have contributed to the occurrence of osteomyelitis in the patient. Factors of trauma and odontogenic also cause osteomyelitis in the patient.

The prognosis of patients with osteomyelitis depends on several factors, including a secondary infection. The secondary infection occurred due to the accumulation of inflammatory exudate in the bone cavity. The patient indicated microcytic hypochromic anemia with suspicion of nutritional and iron deficiency. Iron is essential for the growth and proliferation of bacteria, including those that cause osteomyelitis. Therefore, in chronic osteomyelitis cases, there is often increased demand for iron by bacteria, leading to iron deficiency anemia, which is typically microcytic hypochromic. However, the severity of chronic maxillary osteomyelitis cannot be solely determined by microcytic hypochromic anemia. Other factors, such as the extent of bone involvement, duration of the infection, and underlying medical conditions, such as diabetes, also play a significant role in the severity of the disease. 34 35 Individuals with diabetes may have a compromised vascular supply, leading to tissue ischemia, which impairs the delivery of antibiotics to the site of infection, making it difficult to eradicate the infection. Additionally, individuals with diabetes may have a weakened immune system, making them more susceptible to secondary infections, especially if the initial infection is not adequately treated. 6 22 36 In this patient, the bacteria were confirmed as *Morganella morganii* and *Klebsiella pneumoniae* . These two bacteria are not commonly found in maxillary osteomyelitis. Most of the cases reported the involvement of both bacterial and fungal infections like actinomycosis, 10 11 12 mucormycosis, 13 14 15 16 17 *Escherichia coli* , 37 *Staphylococcus aureus* , 38 *Veillonella species* , 39 *Candida* , 40 and *Kocuria* . 41 The identifying the bacterial and fungal involvement in osteomyelitis is needed to provide definitive therapy on target and reduce the pathogenicity during therapy.

There are three types of osteomyelitis management: antibiotic therapy, surgical therapy, and hyperbaric therapy. Other surgical management options include cauterization, sequestrectomy, debridement, decortication, and jaw resection with reconstruction. 42 Chronic osteomyelitis is difficult to treat using antibiotics and hyperbaric therapy due to the formation of pockets containing dead bone cells and organisms covered with fibrous tissue; so, the treatment becomes ineffective. Removal of bones that have been necrosis is the goal of chronic osteomyelitis therapy; so, invasive procedures must be carried out. 8 32 In this case, extensive sequestrectomy was carried out to remove all infected tissues and necrosis so that the opportunity for vascular reperfusion and drainage could occur in infected areas. 29 Antibiotic therapy is given before and after surgery, which has been adjusted to the sensitivity test of the culture test. The nasal sound that appears after surgery appears because the missing palate bone is wide enough; so, a removable denture is needed for subsequent rehabilitation and long-term control to monitor whether there is a reinfection or unconscious expansion of the infection.

## Conclusion

Comprehensive perioperative management in maxillary osteomyelitis in uncontrolled diabetes mellitus includes sequestrectomy, definitive antibiotic therapy based on culture results, and diabetes regulation to improve the successful management of this case.
